# Development and Characterization of Efficient Cell Culture Systems for Genotype 1 Hepatitis E Virus and Its Infectious cDNA Clone

**DOI:** 10.3390/v15040845

**Published:** 2023-03-26

**Authors:** Putu Prathiwi Primadharsini, Shigeo Nagashima, Toshinori Tanaka, Suljid Jirintai, Masaharu Takahashi, Kazumoto Murata, Hiroaki Okamoto

**Affiliations:** 1 Division of Virology, Department of Infection and Immunity, Jichi Medical University School of Medicine, Shimotsuke, Tochigi 329-0414, Japan; 2 Division of Pathology, Department of Basic Veterinary Medicine, Inner Mongolia Agricultural University College of Veterinary Medicine, Hohhot 010018, China

**Keywords:** hepatitis E virus, genotype 1, serial passages, cell culture system, infectious cDNA clone, replication efficiency, species tropism

## Abstract

Hepatitis E virus (HEV) is a major cause of acute viral hepatitis globally. Genotype 1 HEV (HEV-1) is responsible for multiple outbreaks in developing countries, causing high mortality rates in pregnant women. However, studies on HEV-1 have been hindered by its poor replication in cultured cells. The JE04-1601S strain recovered from a Japanese patient with fulminant hepatitis E who contracted HEV-1 while traveling to India was serially passaged 12 times in human cell lines. The cell-culture-generated viruses (passage 12; p12) grew efficiently in human cell lines, but the replication was not fully supported in porcine cells. A full-length cDNA clone was constructed using JE04-1601S_p12 as a template. It was able to produce an infectious virus, and viral protein expression was detectable in the transfected PLC/PRF/5 cells and culture supernatants. Consistently, HEV-1 growth was also not fully supported in the cell culture of cDNA-derived JE04-1601S_p12 progenies, potentially recapitulating the narrow tropism of HEV-1 observed in vivo. The availability of an efficient cell culture system for HEV-1 and its infectious cDNA clone will be useful for studying HEV species tropism and mechanisms underlying severe hepatitis in HEV-1-infected pregnant women as well as for discovering and developing safer treatment options for this condition.

## 1. Introduction

Hepatitis E virus (HEV) is a single-stranded positive-sense RNA virus with an approximately 7.2 kb genome. It is a member of the family *Hepeviridae*, subfamily *Orthohepevirinae.* This subfamily comprises four genera, including the genus *Paslahepevirus*. Most human infections involve the species *Paslahepevirus balayani* genotypes 1, 2, 3, and 4 and less frequently 7 [1].

The genome contains a short 5′-untranslated region (UTR) with a 7-methylguanosine cap, three open reading frames (ORFs) and a short 3′-UTR terminated by the poly(A) tract [2,3]. ORF1 is translated from genomic RNA, while ORF2 and ORF3 are translated from a subgenomic RNA strand [4,5]. ORF1 encodes a non-structural polyprotein containing multiple functional domains involved in viral replication: methyltransferase (MeT), Y domain, papain-like cysteine protease (PCP), hypervariable region (HVR), X or macro domain, helicase (Hel), and RNA-dependent RNA polymerase (RdRp) [6,7,8,9]. ORF2 encodes the capsid protein, which plays a crucial role during virion assembly and viral attachment to the host cell and is the major target for neutralizing antibodies [10,11]. ORF3 encodes a multifunctional phosphoprotein required for virion egress [12,13,14] and is a functional ion channel acting as a viroporin [15]. ORF4—exclusively present in genotype 1 HEV (HEV-1)—encodes a novel protein identified in the coding sequence of ORF1, is synthesized only under conditions of endoplasmic reticulum (ER) stress, and is short-lived, as it is degraded quickly by the host proteasome [16,17].

HEV infection is distributed globally. Among the four major genotypes infecting humans, HEV-1 and genotype 2 HEV (HEV-2) are restricted to humans and have been responsible for multiple outbreaks in developing countries where the virus is transmitted through drinking contaminated water [18]. Although HEV-1 typically affects people in developing countries, such as South Asia and most countries in sub-Saharan Africa [19], imported infections have been reported in industrialized countries, including Japan [20,21,22]. HEV-1 infection in pregnant women frequently leads to infant mortality or premature delivery. Particularly in the third trimester, HEV-1 infection has been linked to a poor prognosis, where the fatality rate can reach up to 30% [23,24]. Genotype 3 HEV (HEV-3) and genotype 4 HEV (HEV-4), conversely, primarily affect populations in industrialized countries and are mainly transmitted through zoonotic foodborne routes, with less frequent routes including through solid organ transplantation or transfusion of blood products [25].

Most HEV infections are self-limiting but carry a risk of progression to chronic infection in those with an immunocompromised status [26]. Ribavirin has been used to treat certain instances of clinical HEV infection, such as chronic cases (caused by HEV-3, HEV-4, and genotype 7 HEV (HEV-7)) or acute fulminant cases (caused by HEV-1, HEV-3, and HEV-4) [27,28]. However, it has a number of major side effects, such as anemia, and although the latest study does not suggest a clear signal of human teratogenicity for ribavirin, it should be used with caution in pregnant women, a major risk group [29].

The development of cell culture systems for HEV-3 and HEV-4 strains has progressed significantly in recent decades [30,31]. However, HEV-1 replicates poorly in cultured cells due to a lack of an efficient cell culture system [16,32,33,34,35,36], which has hindered research on its life cycle, determinants of its species tropism to humans, and mechanisms underlying its severity in pregnant women, as well as the discovery and development of safer treatment options, particularly for pregnant women with fulminant hepatitis caused by HEV-1 infection.

To overcome this obstacle, we attempted to develop an efficient cell culture system for HEV-1 and to construct an infectious cDNA clone of HEV-1 using the JE04-1601S strain, which was recovered from a Japanese patient with fulminant hepatitis E who contracted HEV infection while traveling to India [37] and has been serially passaged 12 times, as a template. Currently, there are seven subtypes of HEV-1 (1a to 1g) [38]. The strain used in the present study is subtype 1f, which is one of the most common circulating human HEV subtypes in South Asia, including India [39] and Bangladesh [40].

## 2. Materials and Methods

### 2.1. Cell Culture

PLC/PRF/5 (ATCC No. CRL-8024; American Type Culture Collection, Manassas, VA, USA), HepG2/C3A (ATCC No. HB-8065), and A549_1-1H8 (a subclone of A549, No. RCB0098; RIKEN BRC Cell Bank, Tsukuba, Japan) cells were grown in growth medium which consists of Dulbecco’s modified Eagle’s medium (DMEM; Thermo Fisher Scientific, Waltham, MA, USA), supplemented with 10% heat-inactivated fetal bovine serum (FBS) (Thermo Fisher Scientific), at 37 °C in a humidified 5% CO_2_ atmosphere, as previously described [41]. LLC-PK1 (ATCC No. CL-101) cells were grown in medium 199 with Earle’s Salt (Thermo Fisher Scientific) and 2.2 g/l NaHCO_3_, supplemented with 3% heat-inactivated FBS. PK15 (ATCC No. CCL-33) cells were grown in DMEM, supplemented with 10% heat-inactivated FBS and 0.1 mM non-essential amino acid (NEAA) (Thermo Fisher Scientific). IBRS-2 (Cellosaurus, CVCL_4528; Swiss Institute of Bioinformatics, Lausanne, Switzerland) cells were grown in DMEM supplemented with 10% heat-inactivated FBS. For A549_1-1H8, following virus inoculation, growth medium was replaced with maintenance medium (50% DMEM, and 50% medium 199 with Earle’s Salt and 2.2 g/l NaHCO_3_) containing 2% heat-inactivated FBS and 30 mM MgCl_2_. All types of culture media contain 100 U/mL penicillin G, 100 µg/mL streptomycin, and 2.5 µg/mL amphotericin B, except for the medium used in Section 2.11.

### 2.2. Viruses

A serum sample of an HEV-1f strain recovered from a Japanese patient with fulminant hepatitis E who contracted the infection while traveling to India (JE04-1601S, 2.8 × 10^6^ copies/mL; referred to as S5 in [37]) was filtrated through a 0.22 µm microfilter (Millex-GV; Merck Millipore, Darmstadt, Germany), aliquoted as virus stocks, and then stored at −80 °C. Filtrated culture medium containing the cell-culture-adapted HEV-3b strain (JE03-1760F passage 26, JE03-1760F_p26; 1.5 × 10^8^ copies/mL) [42] and filtrated culture medium containing the cell-culture-adapted HEV-4c strain (HE-JF5/15F passage 24, HE-JF5/15F_p24; 2.0 × 10^8^ copies/mL) [31,43] were also utilized in this study.

### 2.3. Virus Inoculation and Serial Passages

Passage 0 (p0) was carried out in a monolayer of PLC/PRF/5 cells using the filtrated JE04-1601S with a viral load of 1.5 × 10^6^ copies/well in a six-well plate (Iwaki, Shizuoka, Japan). Culture medium of p0 was filtrated and then inoculated onto a monolayer of A549_1-1H8 cells in a six-well plate with a viral load of 1.1 × 10^4^ copies/well and was regarded as p1. Serial passages were performed until p12 with an inoculum titer of 1.0 × 10^5^ copies/well (except for p2 and p4 with 4.8 × 10^4^ and 6.8 × 10^4^ copies/well, respectively) (Table 1). The volume of inoculum was 200 µL/well. The general protocol for cell culture is described in Section 2.1.

Inoculation of culture supernatants (JE03-1760F_p26, HE-JF5/15F_p24, or JE04-1601S_p12) was performed in a monolayer of PLC/PRF/5, A549_1-1H8, HepG2/C3A, PK15, IBRS-2, or LLC-PK1 cells in a six-well plate with a titer of 1.0 × 10^5^ copies/well or 1.0 × 10^6^ copies/well, unless otherwise stated. After incubation at room temperature for 1 h, the cells were washed five times with phosphate-buffered saline without Mg^2+^ and Ca^2+^ (PBS[–]), and 2 mL of the respective medium (as described in Section 2.1) was added to each well. The cells were then incubated at 35.5 °C in a humidified 5% CO_2_ atmosphere, as previously described [41]. Every other day, half of the culture medium (1 mL) was replaced with fresh respective medium (as described in Section 2.1).

Inoculum was filtrated through a microfilter with a pore size of 0.22 µm before being inoculated. The collected culture medium was centrifuged at 1300× *g* at room temperature for 2 min, and then the supernatant was stored at −80 °C until use.

### 2.4. Quantification of HEV RNA

Total RNA was extracted from culture supernatants of inoculated or transfected cells using TRIzol-LS reagent (Thermo Fisher Scientific) or from cultured cells using TRIzol reagent (Thermo Fisher Scientific). The quantification of HEV RNA was performed by real-time reverse transcription (RT)-polymerase chain reaction (PCR) using a LightCycler apparatus (Roche Diagnostics KK, Tokyo, Japan) with a QuantiTect Probe RT-PCR kit (Qiagen, Tokyo, Japan), a primer set, and a probe targeting the overlapping region of ORF2 and ORF3, according to the previously described method [44]. The limit of detection by RT-PCR used in the current study is 2.0 × 10^1^ RNA copies/mL.

### 2.5. Immunocapture RT-PCR Assay

The immunocapture RT-PCR assay was performed as described previously with some modifications [44]. Briefly, anti-ORF2 monoclonal antibody (MAb) (H6225) [44] (2 µg/mL) was mixed with protein G magnetic beads (Bio-Rad, Hercules, CA, USA) in PBS(-) containing 0.1% bovine serum albumin (BSA), and then the mixture was rotated at room temperature for 2 h. Membrane-associated particles in the culture supernatants of HEV-1-infected cells were pre-treated with 0.1% sodium deoxycholate (DOC-Na) and 0.1% trypsin at 37 °C for 2 h. After washing the magnetic beads twice with PBS(-) containing 0.1% Tween 20, the treated HEV particles and magnetic beads were incubated in PBS(-) containing 0.1% BSA by rotating at room temperature for 2 h. The supernatants were collected and the magnetic beads were washed three times with PBS(-) containing 0.1% Tween 20. Total RNA in the supernatant and magnetic beads was extracted with TRIzol-LS reagent and TRIzol reagent, respectively, and then subjected to quantification of HEV RNA as described in Section 2.4.

### 2.6. The Determination and Analysis of Full-Length and Partial Genome Sequences of JE04-1601S Strains

The full-length genomic sequences of the JE04-1601S strains (wild-type, p10, and p12) were determined according to the method described previously [42]. In brief, total RNA extracted from a serum sample (for wild-type isolate) or culture medium (for p10 and p12 isolates) was subjected to cDNA synthesis followed by nested PCR of eight overlapping regions including the extreme 5’- and 3’-terminal regions. The amplified regions excluding the primer sequences were nucleotide (nt) 1–132 (132 base pairs (bp)), nt 19–1296 (1278 bp), nt 1058–2094 (1037 bp), nt 2024–3129 (1106 bp), nt 2938–4695 (1758 bp), nt 4598–6376 (1779 bp), nt 6297–7122 (826 bp), and nt 7050–7192 (143 bp); the nt positions are numbered in accordance with the JE04-1601S genome obtained in the present study. The extreme 5’-end sequence (nt 1–132) was determined using the First Choice RLM-RACE kit (Ambion, Austin, TX, USA) [45]. Amplification of the 3′-end sequence (nt 7050–7192 (143 bp): poly(A) tail excluded) was performed in accordance with the previously described method [45]. The amplification product was sequenced on both strands directly or after cloning into the plasmid vector (T-vector pMD20; TaKaRa Bio, Shiga, Japan), using the BigDye Terminator v3.1 Cycle Sequencing Kit on an ABI PRISM 3130*xl* Genetic Analyzer (Thermo Fisher Scientific). The sequence analysis was performed using the Genetyx Mac ver. 22 (Genetyx, Tokyo, Japan).

A portion of the ORF1 region (nt 2651–3200, 550 bp) of the cell-culture-generated variants of JE04-1601S (p0 to p12) was determined. In brief, total RNA extracted from culture medium was reverse-transcribed with SuperScript IV (Thermo Fisher Scientific) and then subjected to PCR in the presence of ExTaq (TaKaRa Bio), using primers HE570 (sense: TGCTTATCGGGAGACTTGC, nt 2632–2650) and HE571 (anti-sense: GTGCTCAAAGTCGATGGCTG, nt 3201–3220). The thermal cycler conditions were 94 °C for 2 min, 35 cycles (94 °C, 30 s; 55 °C, 30 s; 72 °C, 75 s), and then 72 °C for 7 min.

A phylogenetic tree was constructed using the neighbor-joining tree of Jukes–Cantor distances based on the entire genomic sequences of three JE04-1601S strains, all known genotype 1 strains (1a, n = 13; 1b, n = 10; 1c, n = 2; 1d, n = 1; 1e, n = 1; 1f, n = 32; 1g, n = 18; and unclassified subtypes, n = 2) [38], and each one of prototype strains of genotypes 2–8 [38]. Multiple alignments were generated using the MUSCLE software program, version 3.5 [46].

### 2.7. Construction of Full-Length Infectious cDNA Clones

To generate a full-length infectious cDNA clone of JE04-1601S_p12, RNA was extracted from culture medium containing JE04-1601S_p12 using TRIzol-LS. cDNA was synthesized using SuperScript IV (Thermo Fisher Scientific) with primer SSP-T (Table 2). Using the synthesized cDNA as template, three fragments covering the entire JE04-1601S_p12 genome were amplified by PCR with Platinum SuperFi II DNA Polymerase (Thermo Fisher Scientific) (see Figure 4). Fragment 1-1 (F1-1) was amplified with primers 1601S-1 and 1601S-7, fragment 1-2 (F1-2) was amplified with primers 1601S-8 and 1601S-2, and fragment 2 (F2) was amplified with primers 1601S-3 and 1601S-4 (Table 2). The three overlapping amplified fragments were designed to share a 15-nt homologous sequence.

The amplified fragments were purified using a FastGene Gel Extraction Kit (Nippon Genetics Europe, Tokyo, Japan). First, F1-1 and F1-2 were subcloned into pUC19 vector, which harbors the T7 promoter. A cDNA clone of pJE03-1760F [47] used as vector for F1-1 and F1-2 was linearized using inverse PCR in accordance with the previously described method [48] with the pJE03-1760F clone as a template, a high-fidelity DNA polymerase (KOD Plus ver. 2; Toyobo, Osaka, Japan), and primers 1601S-9 and 1601S-6 (Table 2). The amplicons were then purified. F1-1 and F1-2 were fused to generate pJE04-1601S_p12 F1 using the In-Fusion Snap Assembly (TaKaRa Bio), according to the protocols provided by the manufacturer. In brief, an In-Fusion reaction was performed in a total volume of 10 µL, containing 2 µL of 5× In-Fusion Snap Assembly Master Mix, 100 ng of each fragment (F1-1, F1-2, and pUC19 vector), and dH_2_O. The reaction mix was incubated at 50 °C for 15 min and then placed on ice, where 2.5 µL of each mixture was transformed into *Escherichia coli* Stellar Competent Cells (TaKaRa Bio). The plasmids were then extracted, and the F1-1 and F1-2 regions of pJE04-1601S_p12 were sequenced using Sanger’s method described in Section 2.6. Next, the F1 fragment was amplified by PCR, with the pJE04-1601S_p12 F1 as a template, a high-fidelity DNA polymerase KOD Plus ver. 2, and primers 1601S-1 and 1601S-2 (Table 2), followed by purification of the amplicons.

To construct the full-length cDNA clone of pJE04-1601S_p12 under the T7 promoter, purified F1 and F2 were fused using the In-Fusion Snap Assembly. The pUC19 with T7 promoter and poly(A) tract used as a vector was linearized by inverse PCR with the cDNA clone of pJE03-1760F as a template, a high-fidelity DNA polymerase KOD Plus ver. 2, and primers 1601S-5 and 1601S-6 (Table 2). The amplicons were then purified and used for the In-Fusion reaction. The reaction was performed using 200 ng of each fragment (F1, F2, and pUC19 vector), as described above. The plasmids were then extracted, and the sequences of T7 promoter, full-genome, and poly(A) tract were confirmed by Sanger’s method, as described in Section 2.6.

In addition, as a negative control, pJE04-1601S_p12-GAA was generated by mutating the conserved RNA replication motif GDD to GAA (Asp1551Ala [nt A4677C, nt T4678C] and Asp1552Ala [nt A4680C, nt T4681C]). The pJE04-1601S_p12 cDNA clone was used as the template. In brief, two fragments covering the entire JE04-1601S_p12 genome were amplified by PCR with KOD Plus ver. 2. F1 GAA (nt 3384–4686; starting at *Spe*I site to the mutation) was amplified with primers 1601S-*SpeI*-F and 1601S-GAA-R, while F2 GAA (nt 4672–6225; starting at the mutation to *Spe*I site) was amplified with primers 1601S-GAA-F and 1601S-*Spe*I-R. The vector was generated by digesting the pJE04-1601S_p12 clone with *Spe*I-HF (New England Biolabs, Tokyo, Japan) at nt 3395 and nt 6210. The digested cDNA as well as the amplicons (F1 GAA and F2 GAA) were then purified. To generate the full-length cDNA clone of pJE04-1601S_p12-GAA, the three fragments were fused using the In-Fusion Snap Assembly. The reaction was performed using 100 ng of F1 GAA and F2 GAA each and 50 ng of vector, as described above. The sequence between two *Spe*I sites of pJE04-1601S_p12-GAA was confirmed by Sanger’s method, as described in Section 2.6.

### 2.8. In Vitro Transcription and Transfection of RNA Transcripts to PLC/PRF/5 Cells

The full-length cDNA clone and its replication-defective mutant (pJE04-1601S_p12 and pJE04-1601S_p12-GAA, respectively) were each linearized with *Nhe*I (New England Biolabs), and the RNA transcripts were synthesized with T7 RNA polymerase using AmpliScribe^TM^ *T7-Flash*^TM^ Transcription Kit (Epicentre Biotechnologies, Madison, WI, USA). After in vitro transcription, RNA transcripts of the cDNA clones were capped using a ScriptCap m7G Capping System (Epicentre Biotechnologies). The integrity and yield of the synthesized RNAs were determined by agarose gel electrophoresis. An aliquot (2.5 µg) of the capped RNA was transfected into confluent PLC/PRF/5 cells in a well of a six-well plate using the TransIT-mRNA transfection kit (Mirus Bio, Madison, WI, USA) in accordance with the manufacturer’s recommendations. Following incubation at 37 °C for two days, the cells were washed with PBS(-), and then the culture medium was replaced with 2 mL of growth medium, and the cells were incubated at 35.5 °C. Every other day, half of the culture medium (1 mL) was replaced with fresh growth medium. The collected culture medium was centrifuged at 1300× *g* at room temperature for 2 min, and the supernatants were stored at −80 °C until use.

### 2.9. Western Blotting

To detect the expression of ORF2 and ORF3 proteins in the cells transfected with RNA transcripts of pJE04-1601S_p12 and pJE04-1601S_p12-GAA, the proteins in the culture supernatants were separated by sodium dodecyl sulfate-polyacrylamide gel electrophoresis (SDS-PAGE) and blotted onto polyvinylidene difluoride (PVDF) membranes (0.45 µm) (Merck-Millipore), immunodetected with an anti-HEV ORF2 MAb (H6253) [44], or anti-ORF3 MAb (TA0529) [49] and enhanced chemiluminescence HRP-conjugated anti-mouse IgM from goat (Santa Cruz Biotechnology, Santa Cruz, CA, USA), and then visualized by a chemiluminescence assay using SuperSignal West Atto Chemiluminescent Substrate (Thermo Fisher Scientific) with an ImageQuant LAS 500 (GE Healthcare, Turnpike Fairfield, CT, USA), as described previously [47].

### 2.10. Immunofluorescence Assays

HEV-1-infected PLC/PRF/5 cells seeded into eight-well chamber slides (Watson, Tokyo, Japan) were subjected to immunofluorescence staining according to the previously described method [47]. The primary antibody used was anti-HEV ORF2 MAb (H6253) [44] or anti-ORF3 MAb (TA0529) [49], and the secondary antibody was Alexa-Fluor 488-conjugated anti-mouse IgM (Thermo Fisher Scientific). Nuclei were counterstained with 4’,6-diamidino-2-phenylindole dihydrochloride (DAPI, Thermo Fisher Scientific). Slide glasses were mounted with Fluoromount/Plus medium (Diagnostic BioSystems, Pleasanton, CA, USA) and then viewed under an FV1000 confocal laser microscope (Olympus, Tokyo, Japan).

### 2.11. Sensitivity of HEV-1 to Ribavirin in a Cell Culture System

Monolayers of PLC/PRF/5 cells in a 24-well plate were inoculated with 1.0 × 10^5^ copies of cDNA-derived JE04-1601S_p12/well in growth medium without FBS containing 40 or 160 µM ribavirin (Fujifilm Wako, Osaka, Japan) in DMSO (final concentration, 1%) and then subsequently incubated at 37 °C for 2 h. After incubation, the cells were washed five times with PBS(-), and 0.5 mL of growth medium containing 40 or 160 µM ribavirin in DMSO (final concentration, 1%) was added to each well, followed by incubation at 35.5 °C. Every other day, half of the culture medium was replaced with fresh growth medium containing 40 or 160 µM ribavirin in DMSO (final concentration, 1%). The collected culture supernatants were centrifuged at 1300× *g* at room temperature for 2 min, and the supernatants were stored at −80 °C until use. The concentrations of ribavirin used in the current study were determined according to our previous report on the evaluation of drug effect on HEV growth in cultured cells—both concentrations were considered to not cause any significant toxicity—and dose-dependent inhibition on HEV growth was demonstrated [50,51].

### 2.12. Lactate Dehydrogenase (LDH) Cytotoxicity Assay

The cytotoxicity of the drug treatment was quantified by measuring lactate dehydrogenase (LDH) activity released into the culture medium using an LDH cytotoxicity assay kit (Nacalai Tesque, Kyoto, Japan) according to the manufacturer’s protocol. In brief, 100 µL culture supernatants in a 96-well plate were added with 100 µL substrate solution. The plate was protected from light and incubated for 20 min at room temperature. Following the addition of 50 µL stop solution, absorbance was measured at 490 nm using an iMark microplate reader. Measured values were normalized to the value of vehicle control.

### 2.13. Nucleotide Sequence Accession Numbers

The nucleotide sequences of HEV isolates determined in the present study have been deposited in the GenBank/EMBL/DDBJ databases under the following numbers: LC753635 (JE04-1601S), LC753636 (JE04-1601S_p10), LC753637 (JE04-1601S_p12), LC753638 (pJE04-1601S_p12), and LC753639 (pJE04-1601S_p12-GAA).

## 3. Results

### 3.1. Serial Passages of the JE04-1601S Strain

**Figure 1:**
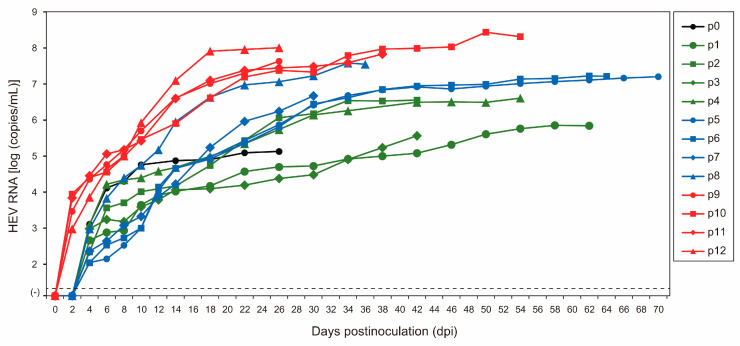
Quantification of HEV RNA in culture supernatants of PLC/PRF/5 (for passage 0, p0) inoculated with serum sample of JE04-1601S/wild-type and in culture supernatants of A549_1-1H8 cells inoculated with culture supernatants of p0, p1, p2, p3, p4, p5, p6, p7, p8, p9, p10, or p11 that were harvested on the final day of each passage (see Table 1). The harvested culture supernatant of each passage was purified by passing through a microfilter with a pore size of 0.22 µm (see Materials and Methods) and then inoculated onto A549_1-1H8 cells. The dotted horizontal line represents the limit of detection by real-time RT-PCR used in the current study, at 2.0 × 10^1^ RNA copies/mL. Each passage was performed for three wells and one representative well showing median viral titer at 10 and 18 days postinoculation was selected, and culture media collected serially from the selected well were subjected to quantification of HEV RNA.

As the initial step in the serial passages of JE04-1601S (p0), a serum sample containing the HEV-1f strain (JE04-1601S_wild-type (wt)) was inoculated onto PLC/PRF/5 cells (Figure 1, Table 1). HEV RNA became detectable in the culture supernatant at 1.3 × 10^3^ copies/mL at 4 days postinoculation (dpi), and its load increased to 1.3 × 10^5^ copies/mL at 26 dpi. The first passage (p1) on A549_1-1H8 cells (Figure 1) was carried out using the culture medium from 26 dpi. The HEV RNA became detectable at 4.6 × 10^2^ copies/mL at 4 dpi and continued to increase, peaking at 6.9 × 10^5^ copies/mL at 62 dpi. Twelve consecutive passages were carried out in A549_1-1H8 cells (Figure 1, Table 1). The time required for the HEV RNA to be detectable in the culture medium shortened during serial passages; up to p8, it started to appear at 4 dpi, whereas from p9 onward, it started to become detectable at 2 dpi. The interval between the inoculation of the cultures and the peak virus titer was shortened as well. During passages, the HEV RNA continued to increase to higher titers in the culture medium, finally peaking at approximately 10^8^ copies/mL from p10 onward. In addition, the yield of the virus also increased during the passages. Collectively, these results suggested that the virus adapted to growth in cell culture. There was no cytopathic effect observed in either PLC/PRF/5 or A549_1-1H8 cells during the serial passages.

**Figure 2:**
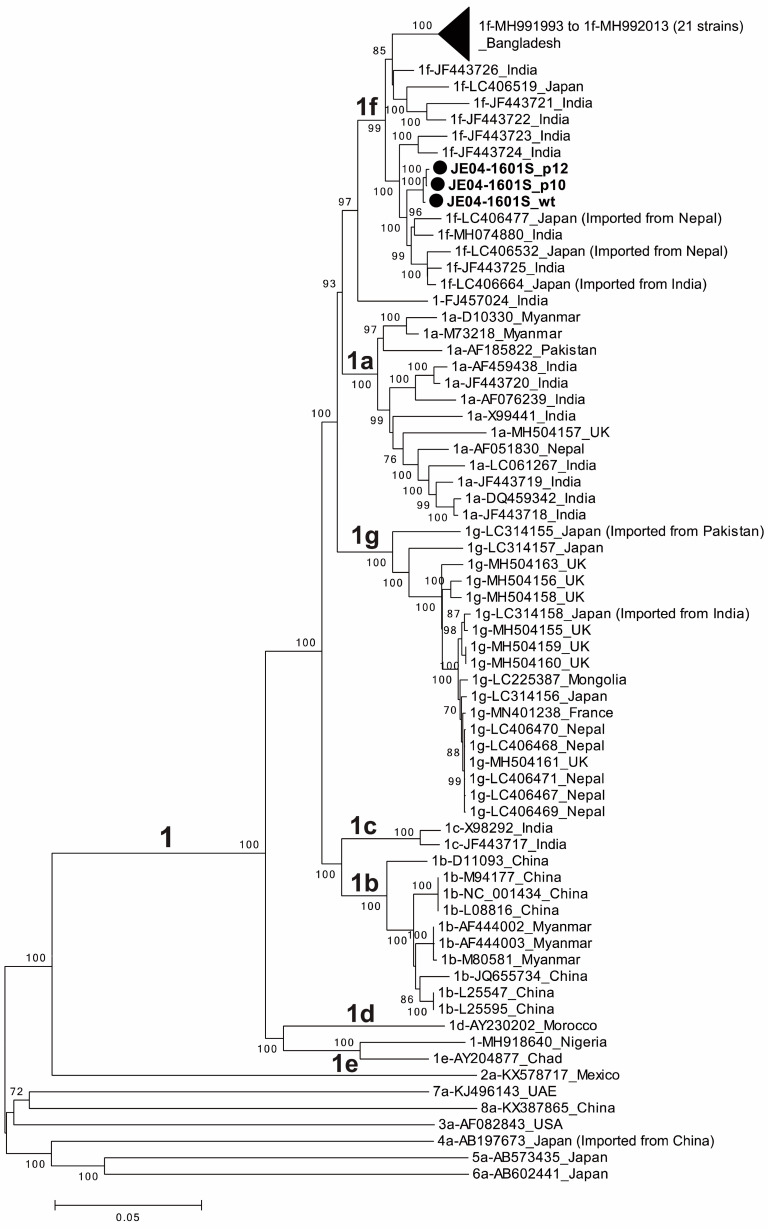
A phylogenetic tree constructed using the neighbor-joining tree of Jukes–Cantor distances based on the entire genomic sequences of three JE04-1601S strains obtained in the present study (JE04-1601S_wild-type (wt), JE04-1601S_p10, and JE04-1601S_p12), all known genotype 1 strains (1a, n = 13; 1b, n = 10; 1c, n = 2; 1d, n = 1; 1e, n = 1; 1f, n = 32; 1g, n = 18; and unclassified subtypes, n = 2), and each one of the prototype strains of genotypes 2–8. The three JE04-1601S strains obtained in the present study are highlighted with closed circles for clarity. Each reference sequence is shown with the genotype/subtype, followed by the accession number and the name of the country in which it was detected. The bootstrap values (≥70%) of the nodes are indicated as a percentage of data obtained from 1000 resamplings. Tips are collapsed for 21 Bangladeshi 1f strains with similar sequences. The scale bar (0.05) represents the number of nucleotide substitutions per site.

A phylogenetic tree was constructed based on the entire genomic sequences of three JE04-1601S strains obtained in the present study (JE04-1601S_wt, JE04-1601S_p10, and JE04-1601S_p12), with all known genotype 1 strains [38] and each one of the prototype strains of genotypes 2–8 [38] (Figure 2). The phylogenetic tree confirmed that the p10 and p12 isolates segregated into a subcluster within subtype 1f together with the wild-type parent.

### 3.2. Mutational Characteristics in Serial Passages of the JE04-1601S Strain

To identify the molecular mechanisms underlying the adaptation of JE04-1601S to growth in cell culture, we determined the complete nucleotide sequence of p10 and p12 as indicated in Figure 2 and compared the sequences of these variants to the sequence of its wild-type parent. Table 3 compares the mutations over the entire genome and the amino acid differences within the three ORFs between the wild-type JE04-1601S and its cell-culture-produced variants. Nucleotide mutations were restricted to ORF1 and ORF2. The p10 isolate had 14 nucleotide mutations, including three mutations at nt 3667, 5090, and 5528 with a mixed nucleotide population of T as well as C that the wild-type virus possessed. A mutation occurred at nt 2988, resulting in an amino acid alteration from Ala to Gly in the helicase region of ORF1. Compared to the p10 isolate, there are nine additional nucleotide mutations in the p12 isolate, where mutations at nt 477 and nt 6613 resulted in amino acid changes from Ser to Phe in the methyltransferase region of ORF1 and from Gly to Ala in ORF2, respectively. Furthermore, one mutation at nt 3667 found in the p10 isolate (mixed nucleotide population of T and C) had a backward mutation to C in the p12 isolate.

The first non-synonymous mutation (Ala to Gly) was observed in the helicase region of ORF1 (nt 2988, aa 988) in the p10 isolate. To determine when it started to occur, the sequences of wild-type JE04-1601S and its cell-culture-generated variants (p0 to p12) within nt 2651–3200 were compared (Table 4). The non-synonymous mutation in the serial passages emerged during p4 and was maintained until p12.

### 3.3. Replication Ability of JE04-1601S_p12 in Various Cell Lines

**Figure 3:**
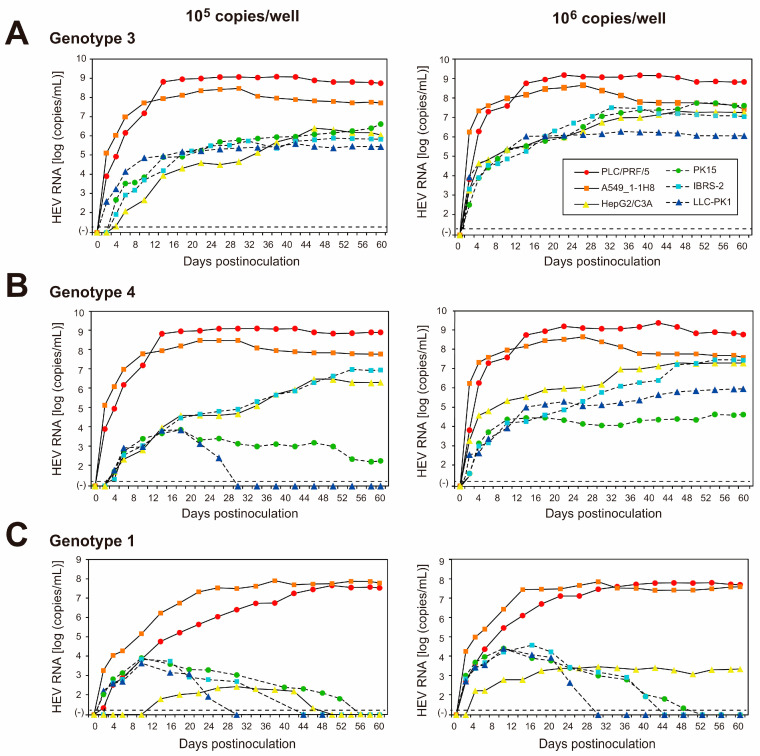
Quantification of HEV RNA in culture supernatants of human-derived cell lines (PLC/PRF/5, A549_1-1H8, and HepG2/C3A cells; indicated with continuous lines) and in culture supernatants of porcine-kidney-derived cell lines (PK15, IBRS-2, and LLC-PK1 cells; indicated with dotted lines) inoculated with the HEV-3 (JE03-1760F_p26) strain (**A**), the HEV-4 (HE-JF5/15F_p24) strain (**B**), or the HEV-1 (JE04-1601S_p12) strain (**C**) at a titer of 1.0 × 10^5^ copies/well (left panels) or 1.0 × 10^6^ copies/well (right panels) in six-well plates. The virus growth was observed for 60 days. The dotted horizontal line represents the limit of detection by real-time RT-PCR used in the current study, at 2.0 × 10^1^ RNA copies/mL. Each inoculation was performed for three wells, one representative well showing median viral titer at 20 and 60 days postinoculation was selected, and culture media collected serially from the selected well were subjected to quantification of HEV RNA.

To examine the replication ability of the cell-culture-generated JE04-1601S_p12 in various cell lines, the culture medium of p12 at 26 dpi (peak HEV RNA titer) was filtered through a 0.22 µm microfilter and then inoculated onto three cell lines of human origin (lung-adenocarcinoma-derived A549_1-1H8 cells and hepatocellular-carcinoma-derived PLC/PRF/5 and HepG2/C3A cells) and three porcine-kidney-derived cell lines (PK15, IBRS-2, and LLC-PK1 cells) at 1 × 10^5^ and 1 × 10^6^ copies/well (Figure 3). The virus growth was observed for 60 days. In addition to the HEV-1 inoculum, inoculation was also carried out using HEV-3 (JE03-1760F_p26) and HEV-4 (HE-JF5/15F_p24) strains for comparison.

The HEV growth was supported in both human- and porcine-derived cell lines inoculated with HEV-3 or HEV-4 at 1 × 10^5^ copies/well (Figure 3A,B, left panels) and 1 × 10^6^ copies/well (Figure 3A,B, right panels). High replication efficiency was observed in both PLC/PRF/5 and A549_1-1H8 cells, where the virus titer in the culture medium peaked at 10^9^ copies/mL for PLC/PRF/5 cells and at 10^8^ copies/mL for A549_1-1H8 cells. Although the virus replication was less efficient in the remaining cell lines, the HEV RNA titer in the culture medium gradually increased, peaking at 10^7^, 10^7^, 10^7^, and 10^6^ copies/mL in HEV-3-inoculated HepG2/C3A, PK15, IBRS-2, and LLC-PK1 cells, respectively. Meanwhile, the HEV RNA titer peaked at 10^7^, 10^4^, 10^7^, and 10^5^ copies/mL in HEV-4-inoculated HepG2/C3A, PK15, IBRS-2, and LLC-PK1, respectively. Only the inoculation of HEV-4 at 1 × 10^5^ copies/well to LLC-PK1 cells was unable to maintain virus replication, as the virus titer in the culture medium started decreasing at 22 dpi and became undetectable from 30 dpi onward. This is possibly dose-dependent, as HEV-4 reached a peak titer of 10^5^ copies/mL when LLC-PK1 cells were inoculated with a titer of 1 × 10^6^ copies/well, and the virus titer in the culture medium was maintained until 60 dpi.

In contrast, the virus replication was only maintained in cell lines of human origin for inoculation with JE04-1601S_p12—albeit with a much lower efficiency in HepG2/C3A cells—where the HEV RNA titer in the culture medium peaked at nearly 10^8^ copies/mL for both PLC/PRF/5 and A549_1-1H8 cells and at 10^3^ copies/mL for HepG2/C3A cells (Figure 3C). Although the HEV RNA was initially detectable at low titer in the culture medium of all porcine-derived cell lines—peaking at approximately 10^4^ copies/mL—it gradually decreased and became undetectable from 30, 44, and 52 dpi onward for LLC-PK1, IBRS-2, and PK15, respectively, despite the fact that the cell-culture-adapted virus was inoculated with a high titer (10^6^ copies/well).

Supporting these results, following the removal of the membrane and ORF3 of HEV particles in the culture supernatants of the human-derived cell lines, nearly 100% of the HEV particles were captured by anti-ORF2 MAb at mid-cultivation (20 days postinoculation) and at the end of cultivation (60 days postinoculation) (Table 5). In contrast, only 10–20% of membrane-unassociated particles were captured by anti-ORF2 MAb at mid-cultivation, and HEV RNA was undetectable at the final cultivation day, in the culture supernatants of infected porcine-derived cell lines (Table 5). This suggested that although HEV-1 might replicate at the initial cultivation days in the porcine-derived cell lines, the replication was not fully supported in these cells.

### 3.4. Construction of an Infectious cDNA Clone of JE04-1601S_p12 and Transfection of Its RNA Transcript to PLC/PRF/5 Cells

**Figure 4:**
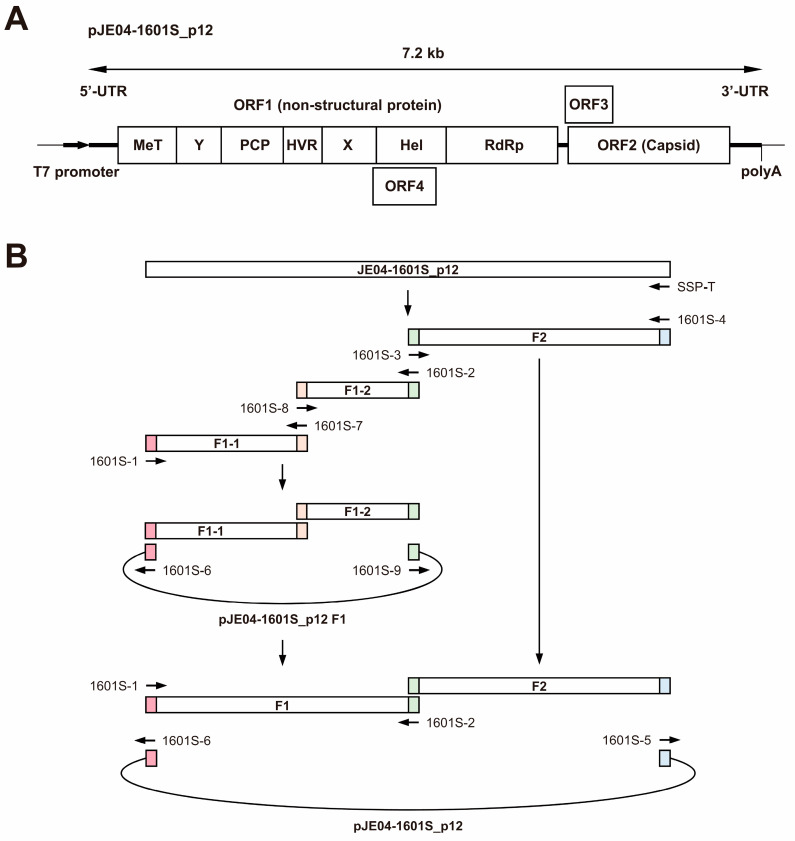
A schematic representation of the full-length genome of the JE04-1601S_p12 strain (**A**) and the strategy to construct its full-length cDNA clone (pJE04-1601S_p12) (**B**). Three fragments covering its whole genome were generated by reverse transcription-polymerase chain reaction (RT-PCR) and then cloned into the pUC19 vector in a stepwise manner using the In-Fusion cloning method. The 15-bp overlaps at their ends are highlighted with same colors. MeT, methyltransferase; Y, Y domain; PCP, papain-like cysteine protease; HVR, hypervariable region; X, X or macro domain; Hel, helicase; and RdRp, RNA-dependent RNA polymerase.

Given the lack of an infectious cDNA clone of HEV-1 with high replication efficiency, JE04-1601S from p12 was used as a template to construct one (Figure 4A). Three fragments (F1-1, F1-2, and F2) covering the whole genome of the JE04-1601S_p12 strain were generated by RT-PCR and then cloned into a pUC19 vector in a stepwise manner according to the In-Fusion cloning method (Figure 4B). Sequence analyses revealed that the resulting infectious cDNA clone had been constructed correctly.

**Figure 5:**
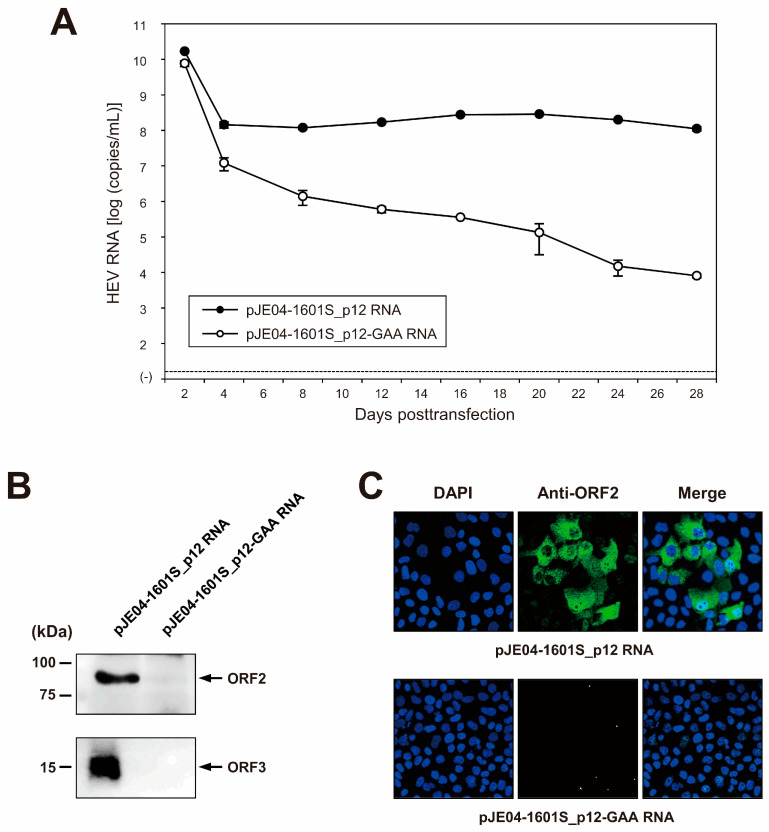
Capability of the cDNA clone of JE04-1760S_p12 to produce infectious progeny viruses. (**A**) Quantification of HEV RNA in culture supernatants. RNA transcript of pJE04-1601S_p12 was transfected to PLC/PRF/5 cells, along with RNA transcript of its replication-defective mutant (pJE04-1601S_p12-GAA), which served as a negative control. HEV growth was observed for 28 days. The data are presented as the mean ± standard deviation (SD) for two wells each. The dotted horizontal line represents the limit of detection by real-time RT-PCR used in the current study, at 2.0 × 10^1^ RNA copies/mL. RNA transfection experiment was performed twice for two wells each, and representative result was shown. (**B**) A Western blot analysis of the culture supernatants transfected with RNA transcript of pJE04-1601S_p12 or that of pJE04-1601S_p12-GAA to examine the expression of HEV ORF2 (upper panel) and ORF3 proteins (lower panel) at day 28 posttransfection. (**C**) Immunofluorescence staining of the cells transfected with the RNA transcript of pJE04-1601S_p12 (upper panel) or that of pJE04-1601S_p12-GAA (lower panel) to examine the HEV ORF2 protein expression at day 28 posttransfection. For Western blotting and immunofluorescence assay, results representative of two experiments are shown.

To examine the capability of the resulting JE04-1601S_p12 cDNA clone to produce a progeny virus, the RNA transcript of pJE04-1601S_p12 was transfected into PLC/PRF/5 cells. To monitor the virus production, the HEV RNA titer in the culture supernatants of the transfected cells was quantified (Figure 5A). The HEV RNA titer gradually increased until 20 days posttransfection (dpt), peaking at 2.9 × 10^8^ copies/mL, and stayed at ~10^8^ copies/mL thereafter. In contrast, a gradual decrease in the HEV RNA titer was observed in the culture supernatants of PLC/PRF/5 cells transfected with the RNA transcript of a replication-defective mutant (pJE04-1601S_p12-GAA), which expressed functionally disrupted RdRp.

Culture supernatants from 28 dpt were then subjected to Western blotting to examine the expression of the viral proteins. The specific bands of ORF2 (Figure 5B, upper panel) and ORF3 (Figure 5B, lower panel) proteins were only detected in the culture supernatants of cells transfected with the RNA transcript of pJE04-1601S_p12 and were undetectable in the culture supernatants of pJE04-1601S_p12-GAA RNA-transfected cells. To examine the intracellular expression of the ORF2 protein, the transfected PLC/PRF/5 cells at 28 dpt were subjected to an immunofluorescence assay (IFA). ORF2 protein was expressed abundantly in the cells transfected with the RNA transcript of pJE04-1601S_p12 (Figure 5C, upper panel), in contrast to the pJE04-1601S_p12-GAA RNA-transfected cells (Figure 5C, lower panel), in which the expression of ORF2 protein was undetectable. Taken together, these results indicate that the HEV-1 cDNA clone is capable of producing an infectious virus.

### 3.5. Characterization of cDNA-Derived JE04-1601S_p12 Progenies in the Cell Culture System

**Figure 6:**
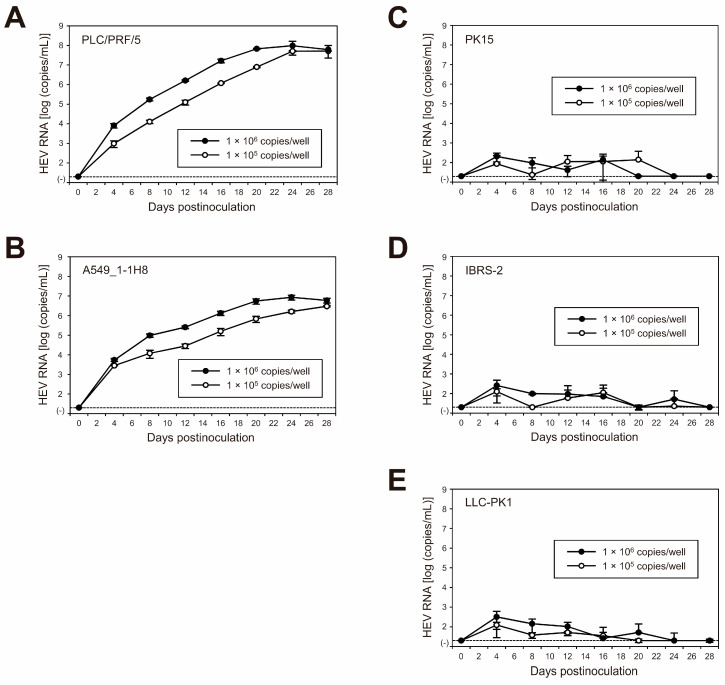
Species tropism of HEV-1 to humans in cell culture of the cDNA-derived JE04-1601S_p12 progeny viruses. Quantification of HEV RNA in culture supernatants of the human-derived cell lines (left panels) PLC/PRF/5 (**A**) and A549_1-1H8 (**B**) cells as well as the porcine-kidney-derived cell lines (right panels) PK15 (**C**), IBRS-2 (**D**), and LLC-PK1 (**E**) cells inoculated with cDNA-derived JE04-1601S_p12 progeny viruses. Inoculum titers were 1.0 × 10^5^ copies/well or 1.0 × 10^6^ copies/well in six-well plates. HEV growth was observed for 28 days. The data are presented as the mean ± SD for three wells each. The dotted horizontal line represents the limit of detection by real-time RT-PCR used in the current study, at 2.0 × 10^1^ RNA copies/mL. Each inoculation was of single experiment for three wells.

To characterize the cDNA-derived JE04-1601S_p12 progenies, they were inoculated onto PLC/PRF/5 (Figure 6A) and A549_1-1H8 (Figure 6B) cells at a titer of 1 × 10^5^ and 1 × 10^6^ copies/well in a six-well plate. During 28 days of observation, the HEV RNA titer in the culture medium of both inoculated cell lines increased gradually and dose-dependently, peaking at 9.7 × 10^7^ copies/mL in PLC/PRF/5 cells and 8.6 × 10^6^ copies/mL in A549_1-1H8 cells, suggesting that the cDNA-derived JE04-1601S_p12 progenies were infectious.

To further examine the species tropism of HEV-1 to humans in the cell culture of the cDNA-derived JE04-1601S_p12 progenies, the progenies were inoculated to the non-human-derived (porcine kidney) cell lines of PK15 (Figure 6C), IBRS-2 (Figure 6D), and LLC-PK1 (Figure 6E). Despite being inoculated with the same titers as in cell lines of human origin (1 × 10^5^ and 1 × 10^6^ copies/well), the genomic RNA of HEV-1 in porcine-derived cell lines was initially detectable at low titer, peaking at approximately 10^2^ copies/mL, then continued to decrease. These results further support the results described in Section 3.3 that the replication of HEV-1 is not fully supported in porcine-derived cell lines.

**Figure 7:**
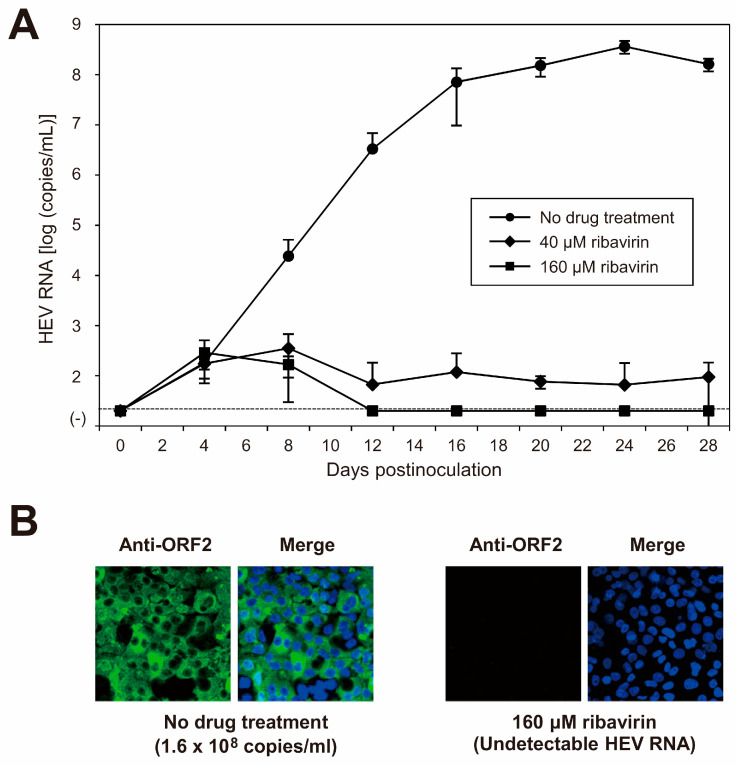
Sensitivity of HEV-1 to ribavirin in the cell culture system. (**A**) Quantification of HEV RNA in culture supernatants of PLC/PRF/5 cells inoculated with cDNA-derived JE04-1601S_p12 progeny viruses (1.0 × 10^5^ copies/well) in the presence of 40 or 160 µM ribavirin in DMSO (final concentration, 1%). HEV kinetics were observed for 28 days. The data are presented as the mean ± SD for three wells each. The dotted horizontal line represents the limit of detection by real-time RT-PCR used in the current study, at 2.0 × 10^1^ RNA copies/mL. The inoculation was of single experiment for three wells. (**B**) Immunofluorescence staining of PLC/PRF/5 cells inoculated with cDNA-derived JE04-1601S_p12 progenies in the presence of 160 µM ribavirin (right panel) to examine the HEV ORF2 protein expression at day 28 postinoculation in comparison to the ORF2 protein expression in untreated control cells (left panel). Results representative of two experiments are shown.

Ribavirin is currently used to treat certain cases of clinical HEV infections, such as cases of chronic or acute fulminant hepatitis. To examine the sensitivity of the cDNA-derived JE04-1601S_p12 progenies to ribavirin, the cDNA-derived JE04-1601S_p12 progenies were inoculated to PLC/PRF/5 cells (1 × 10^5^ copies/well of 24-well plate) in the presence of 40 or 160 µM ribavirin in DMSO (final concentration, 1%). The virus kinetics were then observed for 28 days. The HEV RNA titer in the culture supernatant decreased in a dose-dependent manner. Ribavirin at 40 µM decreased the HEV RNA titer to 9.5 × 10^1^ copies/mL on the final day of observation (28 dpi), and HEV RNA became undetectable from 16 dpi onward in the culture supernatants of the cells treated with 160 µM ribavirin (Figure 7A). In addition, at 28 dpi, HEV RNA was undetectable in the cells treated with 160 µM ribavirin. Supporting this result, at 28 dpi, ORF2 protein expression was undetectable in PLC/PRF/5 cells treated with 160 µM ribavirin (Figure 7B). These results indicated that the sensitivity of HEV-1 to ribavirin was reproducible in the cell culture of cDNA-derived progenies and that the inhibition effect of ribavirin on HEV growth could be monitored long-term in this cell culture system. LDH cytotoxicity assay carried out using culture supernatants of the PLC/PRF/5 cells inoculated with JE04-1601S_p12 progenies and treated with 160 μM ribavirin, from the final day of cultivation (28 dpi) and mid-cultivation (12 dpi)—where the HEV RNA in culture supernatants are still detectable—suggested no significant toxicity caused by the treatment with 160 μM ribavirin at least until 28 days of treatment (Table 6). These results are supported by the IFA image (Figure 7B, right panel) in which no discernible morphological changes are observed on the nuclei.

## 4. Discussion

Infection with HEV-1 is a major public health concern in South Asian countries and African countries [19], with particularly high fatality rates seen in pregnant women [23,24]. In addition to the tendency for HEV-1 infection to cause severe hepatitis in pregnant women, unlike infections caused by HEV-3, HEV-4, HEV-7, or the rat HEV—which are zoonotic—infection with HEV-1 is characterized by its species tropism to humans [52]. Although the exact determinants and mechanisms behind the severity of HEV-1 infection in pregnant women as well as its tropism to humans remain elusive, studies on these subjects—in addition to efforts to search for and develop safer treatment options for pregnant women—have been hampered by the lack of an efficient cell culture system for HEV-1 [16,33]. This obstacle prompted us to attempt to develop such a cell culture system and an infectious cDNA clone of HEV-1 with high replication efficiency.

The HEV-1 strain (JE04-1601S) used in the present study was of subtype 1f (Figure 2) and was recovered from a Japanese patient with fulminant hepatitis E who contracted the infection while traveling to India [37]. Previously, we have reported that two cell lines, PLC/PRF/5 and A549 cells, supported efficient propagation of HEV-3 [41] and HEV-4 [43], and therefore, we selected these two cell lines for the cultivation of HEV-1 as well. Similarly, as shown in Figure 3 in the current study, in the first ten days or so, the HEV RNA titer in the culture supernatants of A549_1-1H8 cells was higher than that in PLC/PRF/5 cells; in addition, the HEV RNA reached the peak level faster, and thus, in order to shorten the time required for consecutive passages, we carried out the passages in A549_1-1H8 cells. Following the initial propagation in PLC/PRF/5 cells, the culture medium containing JE04-1601S_p0 was serially passaged up to 12 times in A549_1-1H8 cells, resulting in markedly increased replication efficiency with no cytopathic effect (Figure 1) and indicating that the virus had adapted to growth in cell culture. These results were consistent with our previous reports on the serial passages of both HEV-3 (JE03-1760F) [42] and HEV-4 (HE-JF5/15F) [43] strains, in which adaptation to growth in cell culture resulted in the shortening of the interval between the inoculation of cultures and maximum virus yield, as well as an increase in the virus yield.

Increased replication efficiency in vitro might be the result of non-synonymous and synonymous mutations that emerged during the serial passages [42,53]. A comparison of the full-genome sequences of JE04-1601S_wt and its variants obtained from cell culture (p10 and p12, Table 2) demonstrated that both non-synonymous and synonymous mutations occurred during serial passages. The first non-synonymous mutation emerged during p4 and was maintained until p12 (Table 3), whereas one of the three mixed nucleotide populations in the p10 strain had a backward mutation in the p12 strain. Although mutations can occur frequently over the entire HEV genome during serial passages for adaptation to growth in cell culture [31,42,53], random mutations might emerge during the passages and only the selected mutations important for the virus are consistently maintained, which can result in viral fitness and thus heightened replication efficiency in vitro [9]. Further studies are warranted to determine which mutations are responsible for the heightened replication by site-directed mutagenesis using a reverse genetic system.

The inoculation of a culture supernatant containing JE04-1601S_p12 to various cell lines demonstrated that while HEV-3 and HEV-4 growth is supported in both human- and porcine-derived cell lines, HEV-1 growth was only fully supported in cell lines of human origin, in which HEV-1 replicated efficiently to reach an HEV RNA titer beyond 10^8^ copies/mL in the culture supernatants of PLC/PRF/5 and A549_1-1H8-inoculated cells, whereas it was not fully supported in porcine-derived cell lines as the replication was only demonstrated at the initial cultivation days (Figure 3 and Table 5), potentially recapitulating the narrow tropism of HEV-1 observed in vivo.

Although HEV-1 can replicate in various cell lines of human origin, the virus titer in the culture supernatant of the infected cells generally peaked at 10^4^ to 10^5^ copies/mL in previous reports by other study groups [16,33,34,35,36,54,55]. However, in the present study, the peak HEV RNA titer reached approximately 10^8^ copies/mL during serial passages (Figure 1), indicating an efficient replication capacity in our cell culture system. In addition, the robust cell culture system for HEV-1 in the present study is also capable of monitoring virus kinetics over a relatively long period, which will be useful for various studies on this genotype, including the evaluation of the efficacy of novel anti-HEV candidates.

Several factors might play role in the efficient replication achieved by the JE04-1601S strain during the passages. In addition to a possible role of adaptive nucleotide mutations, we cannot rule out the possible contribution of the clinical background of the patient from whom the sample originated, as he was suffering from fulminant hepatitis E with a high viral RNA titer in the serum (2.8 × 10^6^ copies/mL). Our previously reported efficient cell culture system for HEV-4 (HE-JF5/15F) [43] was also based on a clinical sample from a patient with fulminant hepatitis E. The contribution of the clinical background to an efficient cell culture system has also been reported for the widely used hepatitis C virus JFH-1 [56], which was isolated from a patient with fulminant hepatitis C.

The potential contribution of differences in the multiplication efficiency of diverse HEV strains in cell culture should also be considered. Recently, it was reported that two mutations in the ORF1 of HEV-1 (Ala317Thr and Val1120Ile) resulted in enhanced virus replication in vitro [33], which may be associated with fulminant hepatitis. The HEV-1 strain used in the present study (JE04-1601S_wt) originally possessed Thr317 and Ile1120. This may also have influenced its higher infective capability than other HEV-1 strains, which might have contributed in part to the efficient replication achieved during serial passages.

Another important factor influencing the efficient replication might be ORF4, which is specific to HEV-1. It is short-lived due to its quick degradation by the host proteasome and is synthesized only under conditions of ER stress [16,17]. ER stress might be an ideal cellular condition for the optimal replication of HEV-1, as can be seen in pregnant women, and can partly explain the tendency for this high-risk group to progress to fulminant hepatitis. Other conditions that induce ER stress can also predispose individuals to develop fulminant-hepatitis-associated HEV-1 infection. In a limited analysis of HEV-1 strains, five out of seven sequences suggested to harbor a proteasome-resistant ORF4 were obtained from fulminant hepatitis patients [16]. Given that the HEV-1 strain used in the present study was obtained from a fulminant hepatitis patient, the expression of ORF4 might have been high, and it might have been proteasome-resistant, thus conferring the ability to replicate efficiently in cell culture. Further analyses using our efficient cell culture system for HEV-1 to confirm this hypothesis are warranted.

Given the absence of an infectious full-length HEV-1 clone with high replication efficiency, we used the cell-culture-adapted JE04-1601S from p12 as the template to construct a full-length cDNA clone using the In-Fusion cloning method (Figure 4). Results from the transfection of its RNA transcript to PLC/PRF/5 cells demonstrated that it successfully produced an infectious virus where the HEV RNA titer in the culture supernatant exceeded 10^8^ copies/mL (Figure 5A), which is around the same as the highest RNA titer observed in the serial passages (Figure 1, p12). The detectable expression of viral proteins by Western blotting (ORF2 and ORF3) in the culture supernatant of the transfected PLC/PRF/5 cells (Figure 5B) and the detectable expression of ORF2 intracellularly by IFA (Figure 5C) further support the notion that the cDNA clone of JE04-1601S_p12 is capable of producing an infectious virus.

The characterization of the cDNA-derived JE04-1601S_p12 progenies demonstrated that the HEV RNA titer in the culture medium of inoculated PLC/PRF/5 (Figure 6A) and A549_1-1H8 (Figure 6B) cells increased gradually and dose-dependently, peaking at nearly 10^8^ copies/mL in PLC/PRF/5 cells and indicating that the cDNA-derived JE04-1601S_p12 progenies were indeed infectious in the cell lines of human origin. In contrast, despite being inoculated with the same titers as in human-derived cell lines, the HEV-1 growth in non-human-derived (porcine kidney) cell lines was only temporary with the HEV RNA titer in the culture medium being initially detectable at low titer, peaking at approximately 10^2^ copies/mL, then continued to decrease thereafter (Figure 6C–E). These results are consistent with the results demonstrated in Figure 3 and Table 5, suggesting that HEV-1 replication is not fully supported in porcine-derived cell lines, further recapitulating the narrow tropism of HEV-1 observed in vivo. Of note, it is likely that the cDNA-derived JE04-1601S_p12 progenies would replicate even more efficiently following serial passages, as evidenced by the serial passages of its wild-type strain.

In terms of the species tropism of HEV-1 to humans as further confirmed in the present study, multiple viral and host factors might play a role in the restricted tropism. Genotype-specific codon usage bias in HEV-1 is generally stronger than that of HEV-3 and HEV-4. HEV-3 and HEV-4 strains derived from either human or swine have more diverse codon usage patterns in their ORFs than HEV-1 [57]. Furthermore, in a correspondence analysis based on relative synonymous codon usage data, HEV genotypes appeared to cluster into HEV-1, and HEV-3 and HEV-4; based on ORF1, HEV-1 is clearly separated from other groups, partially reflecting the fact that HEV-1 infection is restricted to human hosts, while HEV-3 and HEV-4 strains were found in various animal species and were capable of cross-species transmission [58]. Previous attempts have been made to establish HEV-1 infection in non-human hosts (swine) in vitro and in vivo using intergenotypic chimeras [59,60,61,62]. In this strategy, HEV-1 is used as the genomic backbone, and various genomic regions are replaced with the corresponding regions of HEV-3 and HEV-4. However, the intergenotypic chimeras were unable to infect swine either in vitro or in vivo, suggesting that swine cells might lack essential host factors required to establish infection in pigs. In addition, it might reflect the functional importance of species-specific protein–protein interactions during HEV replication [52]. It will be interesting to further elucidate this topic in future studies while taking advantage of the availability of an efficient cell culture system of HEV-1 as well as the cDNA clone with high replication efficiency established in the present study.

Ribavirin is currently used to treat certain cases of clinical HEV infection, such as chronic or acute fulminant cases [27,28]. The further characterization of the cDNA-derived JE04-1601S_p12 progenies revealed that treatment with ribavirin strongly inhibited HEV-1 growth in our cell culture system, with the inhibition maintained over a long period of time (Figure 7). However, although a clear signal of human teratogenicity for ribavirin treatment for pregnant women—where HEV-1 infection leads to mortality in 30% of cases [23]—was not suggested, it has to be administered with caution [29]. The HEV-1 cDNA clone with its high replication efficiency and the robust cell culture system for HEV-1 developed in the present study will be valuable tools for the discovery and development of safer treatment options for this major risk group.

In the present study, we performed only the quantification of HEV RNA in culture supernatants to demonstrate virus growth kinetics. However, since the secreted form of ORF2 protein (ORF2s) is detectable in the culture supernatants of infected cells [63,64], the detection of ORF2s over time would be valuable to monitor virus growth kinetics during propagation at the protein levels. In our lab, we have been using PLC/PRF/5 cells for various HEV experiments, such as to monitor virus growth kinetics during propagation, as well as during the evaluation of drug effect, where we are able to obtain data on HEV replication ability [41,42,43,50,51,53]. Although we consider that the data on PLC/PRF/5 cells should be sufficient enough to draw the present conclusion, it is important to show that the adapted strain also replicates in other hepatoma cell lines which are more commonly used, such as Huh7 or S10-3 cells, in our future study.

In conclusion, we established a robust cell culture system for HEV-1 and an infectious cDNA clone of HEV-1 with high replication efficiency; the successful development of these tools might be the result of the adaptation of the virus to growth in cell culture, which may be attributed to selected mutations emerging during serial passages. In addition, the fact that the serum sample used for the initial passage was obtained from a fulminant hepatitis E patient with a high HEV RNA load (and thus the possible presence of proteasome-resistant ORF4) and that it originally had Thr317 and Ile1120 in the ORF1 region might partially explain its higher infective capability than other HEV-1 strains. Since the strain used here is of subtype 1f and harbors Thr317 and Ile1120—which are two among multiple factors possibly contributing to the high replication efficiency demonstrated in the current study—future work using other HEV-1 subtypes that do not harbor these amino acids shall provide the explanation for this efficiency. JE04-1601S_p12 and its cDNA-derived progenies replicated efficiently in cell lines of human origin but the replication was not fully supported in porcine-derived cell lines, potentially recapitulating the narrow species tropism in vivo. The efficient cell culture system for HEV-1 and its infectious cDNA clone with high replication efficiency established in the present study will be useful for the further elucidation of the determinants of HEV species tropism, the mechanism underlying the development of severe hepatitis in pregnant women infected with HEV-1, and the discovery and development of safer treatment options, particularly for pregnant women with fulminant hepatitis caused by HEV-1 infection.

## Figures and Tables

**Table 1:** Sources and HEV RNA titers of inocula used for serial inoculations onto A549_1-1H8 cells.

Passage	Inoculum	Cells	Viral Load of HEV Inoculated in Each Well (Copies Per Well) ^a^
0	Serum (JE04-1601S_wt)	PLC/PRF/5	1.5 × 10^6^
1	Culture supernatant (26th day after the first inoculation)	A549_1-1H8	1.1 × 10^4^
2	Culture supernatant (62nd day after the second inoculation)	A549_1-1H8	4.8 × 10^4^
3	Culture supernatant (42nd day after the third inoculation)	A549_1-1H8	1.0 × 10^5^
4	Culture supernatant (42nd day after the fourth inoculation)	A549_1-1H8	6.8 × 10^4^
5	Culture supernatant (70th day after the fifth inoculation)	A549_1-1H8	1.0 × 10^5^
6	Culture supernatant (54th day after the sixth inoculation)	A549_1-1H8	1.0 × 10^5^
7	Culture supernatant (64th day after the seventh inoculation)	A549_1-1H8	1.0 × 10^5^
8	Culture supernatant (30th day after the eighth inoculation)	A549_1-1H8	1.0 × 10^5^
9	Culture supernatant (36th day after the ninth inoculation)	A549_1-1H8	1.0 × 10^5^
10	Culture supernatant (26th day after the tenth inoculation)	A549_1-1H8	1.0 × 10^5^
11	Culture supernatant (54th day after the eleventh inoculation)	A549_1-1H8	1.0 × 10^5^
12	Culture supernatant (38th day after the twelfth inoculation)	A549_1-1H8	1.0 × 10^5^

^a^ Quantification of HEV RNA was performed after filtration of the serum sample or the culture supernatant through a 0.22 µm microfilter.

**Table 2:** The primers used to construct the pJE04-1601S_p12 and pJE04-1601S_p12-GAA clones.

Name	Polarity	Sequence (5′ to 3′)	Note
SSP-T		AAGGATCCGTCGACATCGATAATACGTTTTTTTTTTTTTTT	cDNA synthesis
1601S-1	+	GCTTAATACGACTCACTATAGCAGACCACATATGTGGTCGATGCC	T7 promoter (underlined) and positive-strand sequence (nt 1–25 ^a^)
1601S-2	–	TTGCATCGGAGATGCCCACCTCG	Negative-strand sequence (nt 3598–3620)
1601S-3	+	GGTGGGCATCTCCGATGCAATCG	Positive-strand sequence (nt 3601–3623)
1601S-4	–	GCCCCAAGGGGTTATGCTAGTTTTTTTTTTTTTTTTTTTTTTTTTTTTTTTCAGGGAGCGCGAAACGCAGAAAAGAG	pUC19 vector, poly(A), and negative-strand sequence (nt 7167–7192)
1601S-5	+	CTAGCATAACCCCTTGGGGCCTC	pUC19 vector
1601S-6	–	TATAGTGAGTCGTATTAAGCTTGGCG	T7 promoter (underlined) and pUC19 vector
1601S-7	–	ACAGAATGGATTGGCCGACTCCC	Negative-strand sequence (nt 2088–2110)
1601S-8	+	AGTCGGCCAATCCATTCTGTGGC	Positive-strand sequence (nt 2091–2113)
1601S-9	+	GGTGGGCATCTCCGATGCAACTCTAAACGGGTCTTGAGGGG	Positive-strand sequence (nt 3601–3620) and pUC19 vector
1601S-*Spe*I-F	+	TTGGGCAGAAACTAGTGTTCACCC	Positive-strand sequence (nt 3384–3407), *Spe*I site (underlined)
1601S-GAA-R	–	ATCGAGGCGGCACCTTTGAAGGCAGCCACCTGC	Negative-strand sequence (nt 4654–4686), mutated nucleotide (underlined)
1601S-GAA-F	+	AGGTGCCGCCTCGATAGTGCTTTGCAGTGAGTACC	Positive-strand sequence (nt 4672–4706), mutated nucleotide (underlined)
1601S-*Spe*I-R	–	CGCCATTAGTACTAGTAAAATAAAGATCC	Negative-strand sequence (nt 6197–6225), *Spe*I site (underlined)

^a^ The nucleotide positions are numbered in accordance with the JE04-1601S strain obtained in the present study.

**Table 3:** A comparison of the sequence of the original JE04-1601S strain in serum and its cell-culture-produced variants over the entire genome.

Nucleotide Position	Region (Domain) ^a^	Nucleotide ^b^			Amino Acid	
		JE04-1601S_wt	Passage 10 (p10)	Passage 12 (p12)	Position	Substitution
244	ORF1 (MeT)	C	T	T	73	-
433	ORF1 (MeT)	C	C	T	136	-
477	ORF1 (MeT)	C	C	T	151	Ser to Phe
617	ORF1 (MeT)	C	T	T	198	-
895	ORF1 (Y)	A	A	G	290	-
1270	ORF1 (Y)	C	T	T	415	-
1738	ORF1 (PCP)	C	T	T	571	-
1870	ORF1 (PCP)	T	C	C	615	-
2311	ORF1 (HVR)	C	T	T	762	-
2356	ORF1 (X)	T	C	C	777	-
2728	ORF1 (X)	C	T	T	901	-
2988	ORF1 (Hel)	C	G	G	988	Ala to Gly
3112	ORF1 (Hel)	T	C	C	1029	-
3298	ORF1 (Hel)	T	T	C	1091	-
3667	ORF1 (RdRp)	C	Y^b^	C	1214	-
4417	ORF1 (RdRp)	T	C	C	1464	-
4603	ORF1 (RdRp)	T	T	C	1526	-
5090	ORF1 (RdRp)	C	Y	T	1689	-
5528	ORF2	C	Y	T	128	-
6613	ORF2	G	G	C	490	Gly to Ala
6770	ORF2	C	C	T	542	-

^a^ MeT, methyltransferase; Y, Y domain; PCP, papain-like cysteine protease; HVR, hypervariable region; X, X or macro domain; Hel, helicase; RdRp, RNA-dependent RNA polymerase. ^b^ Y = U/C.

**Table 4:** A comparison of the sequences of wild-type JE04-1601S and its cell-culture-generated variants (p0 to p12) within nt 2651–3200.

Wild-Type or Variants of JE04-1601S	Nucleotide Position		
	2728	2988	3112
Wild-type (serum)	C	C	T
p0 (culture supernatant, 26 dpi ^a^)	C	C	C
p1 (culture supernatant, 62 dpi)	T	C	C
p2 (culture supernatant, 42 dpi)	T	C	C
p3 (culture supernatant, 60 dpi)	T	C	C
p4 (culture supernatant, 70 dpi)	T	S ^b^	C
p5 (culture supernatant, 54 dpi)	T	G	C
p6 (culture supernatant, 64 dpi)	T	G	C
p7 (culture supernatant, 30 dpi)	T	G	C
p8 (culture supernatant, 36 dpi)	T	G	C
p9 (culture supernatant, 26 dpi)	T	G	C
p10 (culture supernatant, 54 dpi)	T	G	C
p11 (culture supernatant, 38 dpi)	T	G	C
p12 (culture supernatant, 26 dpi)	T	G	C

^a^ dpi, days postinoculation. ^b^ S = G/C.

**Table 5:** Reactivity of anti-ORF2 with membrane-unassociated JE04-1601S_p12 (HEV-1) particles as evaluated by immunoprecipitation and real-time RT-PCR.

Inoculated Cells	% of Captured HEV Particles ^a^ in the Total HEV-1 Per Tube	
	20 Days Postinoculation	60 Days Postinoculation
PLC/PRF/5	98.7	95.8
A549_1-1H8	98.9	98.5
HepG2/C3A	95.6	100.0
PK15	10.1	N/A ^b^
IBRS-2	12.3	N/A
LLC-PK1	20.5	N/A

^a^ Membrane-unassociated HEV particles generated from the culture supernatants of JE04-1601S_p12-infected cells following treatment with 0.1% sodium deoxycholate (DOC-Na) and 0.1% trypsin at 37 °C for 2 h were subjected to immunoprecipitation, and the precipitates were then subjected to real-time RT-PCR. ^b^ Not applicable (HEV RNA was undetectable in culture supernatants).

**Table 6:** Lactate dehydrogenase (LDH) released into the culture supernatants of PLC/PRF/5 cells inoculated with JE04-1601S_p12 progenies and treated with ribavirin.

Treatment	LDH Release (Mean ± SD) ^a^	
	12 Days Postinoculation	28 Days Postinoculation
No drug treatment	2.4% ± 0.3%	2.4% ± 0.3%
40 μM Ribavirin	2.1% ± 0.4%	2.4% ± 0.3%
160 μM Ribavirin	2.8% ± 0.3%	3.7% ± 0.3%

^a^ LDH release was determined in the culture supernatants from the final day of cultivation (28 days postinoculation) in comparison to that at 12 days postinoculation (mid-cultivation) to examine the cytotoxicity caused by the ribavirin treatment. Data represent the mean ± SD of triplicate wells.

## Data Availability

All data are presented in the manuscript.
